# How common are vascular injuries in open tibial fractures? A prospective longitudinal cohort study

**DOI:** 10.1007/s00590-019-02416-4

**Published:** 2019-03-13

**Authors:** O. O’Malley, A. J. Trompeter, S. Krishnanandan, M. Vesely, P. Holt, G. Goh, N. Papadakos, V. Bhatia, C. B. Hing

**Affiliations:** 1 Department of Trauma and Orthopaedics, St George’s University Hospitals NHS Foundation Trust, London, UK; 2 Department of Radiology, The Alfred Hospital, Melbourne, Australia; 3 St George’s University London, St George’s University Hospitals NHS Foundation Trust, London, UK

**Keywords:** Tibia, Angiogram, Vascular injury

## Abstract

**Background:**

Tibial fractures have an incidence of 15% of all adult fractures. They have been shown to have the highest incidence of non-union in long bone fractures and the highest incidence of vascular injury. Evidence from the literature suggests that a good vascular supply is important to ensure bone union. The aim of our study was to prospectively assess the incidence of vascular injuries in open tibial fractures and determine whether they were associated with an increased risk of non-union.

**Methods:**

We performed a prospective study to investigate the incidence of arterial injuries with computed tomography angiography (CTA) in patients with Gustilo–Anderson grade I–III open tibial fractures between 2013 and 2015. CTA was performed with the trauma series at acute admission and reported by two independent musculoskeletal radiologists. Patients were followed up with clinical and radiographic assessment for 1 year.

**Results:**

We recruited 77 patients into the study, and 56 patients (47 males, 9 females) were available for the final analysis, between 16 and 90 years of age. At the initial assessment, 29% had signs of arterial injury with active extravasation in 5%. The most common site of injury was in the diaphysis (87.5%), and the commonest mechanism was a road traffic accident. We found no significant relation between occult vascular injury and non-union (*p* > 0.05).

**Conclusion:**

The incidence of vascular injury in open tibial fractures is 29%, and CTA is therefore a useful test in identifying vascular injuries that may require vascular intervention.

## Introduction

Tibial shaft fractures have an incidence of 15% of all adult fractures and are high-energy injuries often associated with significant soft tissue injury [1]. The main aims of treatment are to restore alignment of the tibia with adequate stability to allow bone union and rehabilitation of the limb. In the United Kingdom (UK), treatment follows the British Orthopaedic Association and British Association of Plastic, Reconstructive and Aesthetic Surgeons guidelines of a combined orthoplastic approach to management in regional specialist centres [2, 3].

Evidence from the literature suggests that a good vascular supply is important to ensure bone union with vascular compromise being implicated in non-union [4–7]. Tibial fractures have been shown to have the highest incidence of non-union of all long bone fractures as well as the highest incidence of vascular injury [8–10]. The incidence of vascular injury associated with type III tibial fractures has been quoted as 9% [11].

Diagnosis of vascular injury at the initial assessment relies on clinical examination with palpable pulses inferring intact vascular supply. However, in the absence of obvious signs of vascular compromise and critical limb ischaemia, vascular injuries may be easily missed yet controversy exists as to whether routine investigation or exploration of open tibial fractures with palpable pulses is justified [12, 13]. It may be difficult to appreciate the degree of internal soft tissue injury on the initial inspection of wound size and imaging, including the presence or absence of vascular injury. However, a recent systematic review of the literature has shown that computed tomography angiogram (CTA) should be the investigation of choice in patients with suspected vascular injury in any anatomic region [14]. CTA is the examination of choice due to its short acquisition time and high diagnostic accuracy. Studies have shown the specificity and sensitivity to be 98.7–100% and 90–95.2%, respectively, for CTA diagnosis of arterial damage in the extremities following trauma. Signs eliciting vascular injury include active extravasation of contrast material, incidence of pseudoaneurysm formation, abrupt narrowing of an artery, loss of opacification of a segment of an artery and arteriovenous fistula formation [15, 16].

The aim of this study was to prospectively elucidate the incidence of vascular injuries associated with open tibial fractures and determine whether vascular injury correlated to an increased risk of non-union.

## Methods

We conducted a prospective longitudinal cohort observational study to assess the incidence of vascular injury in a consecutive series of patients admitted to a level 1 trauma centre in the United Kingdom (UK) with open tibia fractures between April 2013 and November 2015. All patients admitted directly or as part of a tertiary referral pathway were invited to participate in the study. Ethics approval was obtained from the local Research Ethics Committee (IRAS 95683, REC 12/LO/1646). Patients were invited to participate if they had an open fracture of the tibial shaft, plateau or pilon, were greater than 16 years of age and could give informed consent (Table 1). Patients were excluded if they were unable to give consent or follow the study protocol, had a previous vascular injury or had an ipsilateral limb injury including a vascular injury.

**Table 1:** Inclusion and exclusion criteria

Inclusion criteria	Exclusion criteria
Open tibial fractures	Ipsilateral limb injury with vascular injury
> 16 years	Previous vascular injury
Conscious or unconscious	Ankle fractures
Single limb or polytrauma	Unable to consent

All patients enrolled into the study were managed as per the standard British Orthopaedic Association and British Association of Plastic, Reconstructive and Aesthetic Surgeons (BOAST/BAPRAS 4 Algorithm) guidelines from 2013 [2]. The patients received joint care from an orthopaedic and plastic surgeon, and surgical wound debridement and operative fracture stabilisation were performed within 24 h and definitive soft tissue cover within 72 h.

At the initial assessment, general demographics (age, gender and smoking) were recorded together with the injury mechanism, fracture classification, soft tissue injury classification (Gustilo and Anderson [17]) and details of the initial surgical management were recorded.

All patients recruited into the study received a CTA at the time of a trauma series CT scan in the emergency department (ED). This was done within 60 min as per NHS England guidance [18]. At 4 ml/s, 90 ml Omnipaque 300 was used as the contrast medium, and the scan was performed from L2 to the feet in arterial phase. The CTA required an additional 3.6 mSv of radiation exposure in addition to the trauma series CT. Two independent radiologists reviewed each CTA.

All patients received standard routine postoperative management including physical therapy, occupational therapy and psychological support as per their injury, their means of skeletal fixation and their associated injuries.

The primary outcome was the incidence of vascular injury as reported by CTA. Secondary outcomes included non-union in subjects with open fractures and concomitant vascular injury noted on CTA. Time to union was defined by the radiographic union score in the tibia (RUST) score at 6 months [19]. All patients were independently reviewed in a research clinic at 2 weeks, 6 weeks, 3 months, 6 months and 1 year from injury using the SF-36 and visual analogue scale for pain (VAS) scores [20, 21].

Data were collected, anonymised and entered into a standardised spreadsheet (MS Excel, Microsoft, Washington, USA). Basic demographic data were recorded, and incidence of vascular injury was calculated. The association between vascular injury and non-union was analysed with chi-squared testing using SPSS statistics package (IBM Corp. Released 2013. IBM SPSS Statistics for Windows, version 21.0. Armonk, NY).

A significant difference was defined by *p* < 0.05.

## Results

Between 2013 and 2015, 78 patients were recruited into the study and after exclusions and withdrawals, the total number of patients available for the final analysis was 56 (Table 2). All patients included in the study had palpable pulses at admission. All patients had the initial debridement within 24 h and had soft tissue cover within 72 h as per BOAST guidelines.

**Table 2:** Patient demographics

Age (years)	
Range	16–90
Mean	41
Gender	
Male (%)	47 (84%)
Female (%)	9 (16%)
Mechanism of injury	
Road traffic accident (RTA)	31
Fall	18
Other (sports etc.)	5
Not specified	2
Length of hospital stay (days)	
Range	3–63
Mean	15
AO classification	
42-A1	8
42-A2	7
42-A3	4
42-B1	2
42-B2	5
42-B3	5
42-C2	8
42-C3	9
43-A1	1
43-A3	2
43-B1	1
43-B2	1
43-C2	1
43-C3	2
Gustilo–Anderson grade	
3a	23 (41.1%)
3b	17 (30.3%)
3c	16 (28.6%)

The incidence of vascular injury on CTA was 29% (16 patients). A summary of the patients with vascular injury on CTA is found in Table 3. Those with abnormal CTA had a variety of fracture patterns, but a predominance for the diaphyseal region (87.5%) and the predominant mechanism of injury was a high-velocity incident.

**Table 3:** Abnormal CTA patients

Age (years)	AO	Injury	Artery injured	Type of injury	Union
38	42-C2	Hit by train	ATA^b^	Occlusion at fracture level	Non-union
90	43-B2	Fall	ATA and PTA^c^	Occluded proximally athersclerosis	Deceased
30	42-C2	RTA^a^	PTA	Vessel contusion/spasm	Union
51	42-C2	Not given	ATA	Filling defect distal to fracture, complete effacement at distal fracture line	Non-union
41	42-A1	Fall from horse	ATA	Not visible distal to fibular fracture	Union
52	42-A2	RTA	ATA and peroneal	Opacify for segment at open fracture flow reconstitutes distally	Union
53	42-A2	RTA	ATA	Spasm	Union
20	42-B2	RTA	Peroneal	Loss of opacification at fracture level with extravasation	Union
41	42-B2	RTA	ATA	Loss of opacification at fracture level	Union
36	42-C3	RTA	Dorsalis pedis and common peroneal	Do not opacify below fracture	Union
32	42-C2	Fall	ATA	Loss of opacification distal to fracture possible due to spasm	Union
26	42-C3	RTA	ATA	Active extravasation and haematoma	Union
24	42-A1	RTA	PTA	Occlusion	Union
17	43-C3	RTA	ATA	No opacification distal to fracture possible spasm	Union
40	42-A1	RTA	ATA	Extravasation at level of fracture	Union
50	42-B3	RTA	Peroneal	Non-specified injury	Union

^a^*RTA* road traffic accident

^b^*ATA* anterior tibial artery

^c^*PTA* posterior tibial artery

**Fig. 1:**
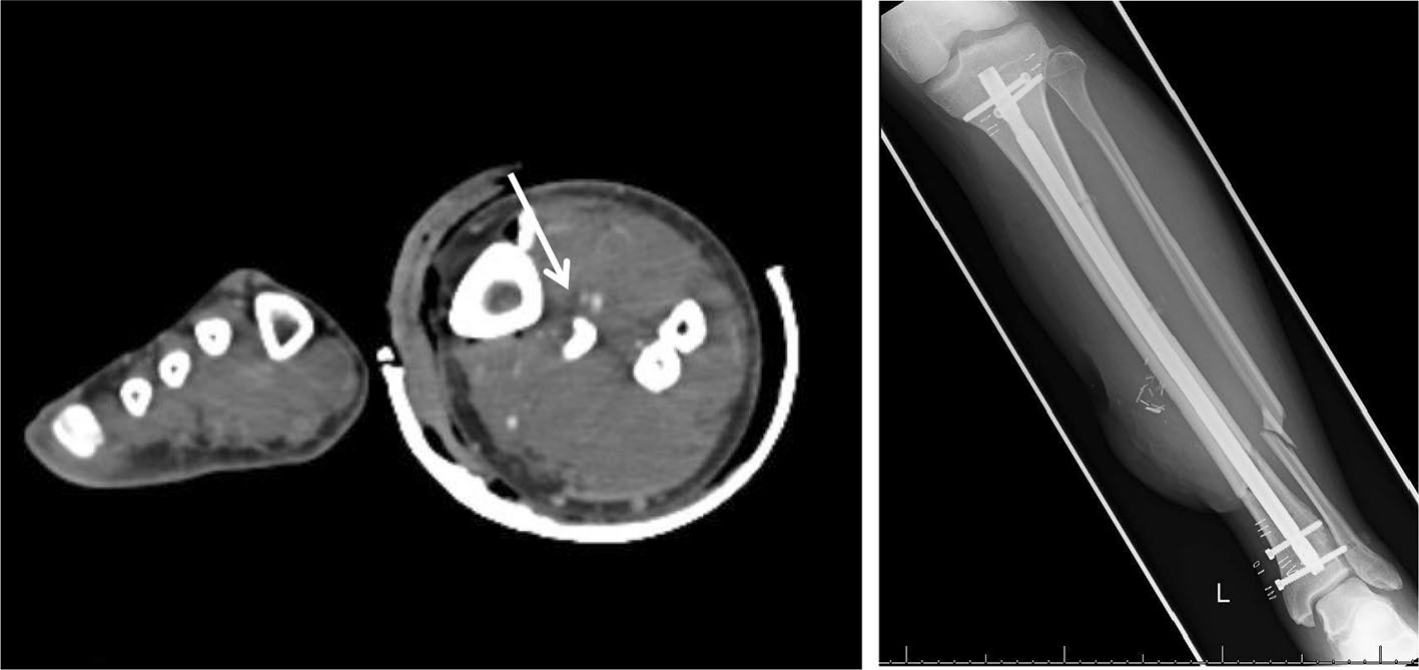
Radiograph of an open tibial fracture and CTA showing associated vascular injury to anterior tibial artery

The anterior tibial artery (ATA) was the most commonly affected artery on CTA reported in 11 out of 16 cases of vascular injury (69%) (Fig. 1). The most common finding being the fracture displacing bone lying in close association to the artery results in attenuated flow. Two ATA injuries involved active extravasation. The peroneal artery was affected in four cases with one case of active extravasation. The posterior tibial artery was injured in three cases and the dorsalis pedis in one case. In all cases of extravasation, road traffic accident (RTA) was the mechanism of injury.

### Bone union

There were four patients (seven percentage) who had non-union and required further surgery as shown in Table 4. Two of these patients did not have a vascular injury and two had a vascular injury. Both patients who had a vascular injury had injuries to the anterior tibial artery. All of the original injuries that led to non-union were in the diaphysis with 75% being multi-fragmentary fractures. With a chi-squared statistic of 1.12, there was no relation between vascular injury and non-union.

**Table 4:** Cases of non-union

Age (years)	Mechanism	Gustilo	Anderson	Vascular injury
38	Train	3c	42-C2	Y^b^
51	Unknown	3c	42-C2	Y
54	RTA^a^	3b	42-C3	N^c^
64	Fall	3b	42-A1	N

^a^*RTA* road traffic accident

^b^*Y* yes

^c^*N* no

### Outcome scores

The mean VAS at 6 weeks was 3, at 3 months was 4.2, at 6 months was 3.9 and at 1 year was 3.4. Pain scores generally decreased overtime as would be expected; however, a lower mean pain score was recorded at the initial follow-up at 6 weeks. In those with vascular injuries, mean VAS scores were higher at each time interval 4.3, 5.9, 4.5 and 4.6, respectively. This indicates higher pain scores in those with a vascular injury, and this could be attributed to severity of their injury.

SF-36 was used as a measure of health-related quality of life and again was recorded at 6 weeks, 3 months, 6 months and 1 year with 91, 93, 95 and 102 as the mean results, respectively. This showed that patients felt their quality of life improved as time progressed as to be expected. In those with vascular injury, mean scores were 89, 96, 88 and 104.5, respectively. This showed a general increase in quality of life with an anomaly at 6 months. Quality of life was lower in those in vascular injury at 5 weeks and 6 months compared to those without, but was higher after 1 year.

## Discussion

We found the incidence of vascular injury in open tibial fractures is to be 29%. This study is the first to prospectively assess the incidence of vascular injury with CTA in lower limb open fractures. Previous literature on concomitant vascular injury in open tibial fractures does not assess incidence directly but rather outcomes of the fracture [9].

The incidence of vascular injury in our study was higher than those previously described; however, only three cases involved 5% active extravasation [5, 11]. This may relate to a small vessel, which has no clinical significance on healing and recovery as demonstrated by these causes going on to uncomplicated union. Furthermore, many of the vascular injuries described were reversible when fracture had been reduced, and therefore, if the CTA was preformed post-fracture stabilisation the incidence of vascular injury may have been lower.

Palpable pulses were felt in all patients on admission; however, 29% were discovered to have vascular injury. Although immediate vascular intervention was not required, the findings would alert physicians to potential need for intervention and ongoing surgical planning.

In terms of the pattern of vascular injury found, the anterior tibial artery was most commonly affected which is similar to previous literature [5, 22–24]. This could be assumed as a result of anterior force commonly resulting from an RTA. We found high-impact injuries such as RTA to be the most common cause of vascular injury, which agrees with previous literature [8].

CTA has previously been proposed as the investigation of choice for suspected arterial injury in trauma. It is quick and readily available, with relatively few contraindications or complications. We had no adverse effects or extra morbidity from the additional angiography sequence in our series, and this correlates with other studies [16]. The vast majority of trauma patients have a CT head to pelvis, and therefore, angiography may be suitable with little extra ionising radiation or risk.

A previous study concluded that routine use of CTA in lower extremity fracture was not indicated. However, the presence of open fracture, distal or shaft tibial fractures increases the risk of having vascular injury on CTA. They also concluded that all their patients who had diminished or absent pulses required vascular treatment and therefore emphasise how important clinical examination is [16].

The literature explains how early exploration and appropriate surgery may lessen the need for amputation in those with vascular injury. The exact nature of the vascular injuries must be detected promptly to minimise the length of ischaemia and revascularisation should be carried out whenever it is possible [13]. The type of vascular injury can also predict the risk of reconstructive complications [24]. Therefore, early recognition of a vascular injury using CTA can aid prompt surgical management and follow-up planning.

In terms of our secondary outcome, non-union in vascular injury, we found no association. This contradicts a previous study that found those with vascular occlusion had a significantly greater incidence of delayed union or non-union [6].

A previous study found a significant relationship between posterior tibial artery injury and non-union; none of the cases identified in our study with posterior tibial artery injury had non-union. Of our non-unions, all were in the diaphysis and 75% were multi-fragmentory, which fits with the results of a previous study [9].

The literature is limited in terms of incidence of vascular injury in open tibial fractures and its direct association with non-union. Although this is the first study to look prospectively at vascular injury in open fractures, it would be useful to gain a larger sample of those with vascular injuries to further assess their outcomes in terms of union. Retrospective studies with larger sample sizes showed poorer outcomes and statistically significant rate of non-union in those with vascular injury [4, 5].

In conclusion, CTA is useful in detecting potential vascular injury; although many may not require vascular intervention, it alerts physicians to those who may require it. CTA should be considered in all those with high-velocity injuries resulting in an open tibial fracture. It is also valuable in preoperative planning for soft tissue reconstructive surgery in order to identify suitable vessels for local or free flaps.
